# Differences in Local and Systemic TFV PK Among Premenopausal Versus Postmenopausal Women Exposed to TFV 1% Vaginal Gel

**DOI:** 10.1097/QAI.0000000000001648

**Published:** 2018-02-07

**Authors:** Andrea Ries Thurman, Neelima Chandra, Nazita Yousefieh, Thomas Kimble, Sharon M. Anderson, Mackenzie Cottrell, Craig Sykes, Angela Kashuba, Jill L. Schwartz, Gustavo F. Doncel

**Affiliations:** * CONRAD, Eastern Virginia Medical School, Norfolk and Arlington, VA; and; † University of North Carolina, Chapel Hill, NC.

**Keywords:** tenofovir pharmocokinetics, HIV, menopause, cervicovaginal, hormones

## Abstract

**Objective:**

We describe and compare the local and systemic pharmacokinetics (PK) of tenofovir (TFV) and TFV-diphosphate (TFV-DP) in healthy premenopausal (PRE) and postmenopausal (POST) women using TFV 1% gel and correlate local PK with other mucosal end points.

**Methods:**

PRE (n = 20) and POST (n = 17) women used 2 doses of TFV 1% vaginal gel, separated by 2 hours. Blood and cervicovaginal samples were obtained 3 and 23 hours after the second dose. PRE women used gel in the follicular and luteal phases of the menstrual cycle. POST women used gel at baseline and again after approximately 2 months of treatment with 0.01% vaginal estradiol (E2) cream.

**Results:**

Median TFV concentrations in cervicovaginal aspirate (ng/mL) and vaginal tissue (ng/mg) were significantly higher in PRE (4.3E10^6^, 49.8) versus POST women (2.6E10^6^, 2.2). POST women had significantly higher median molecular ratios of TFV-DP to TFV (3.7%) compared with PRE (0.19%). After vaginal E2 treatment, the local and systemic PK end points in POST women were generally similar to PRE women (all *P* values > 0.05). Importantly, median vaginal tissue TFV-DP concentrations (fmol/mg) among PRE, POST, and POST women after E2 therapy were similar (292.5, 463.3, and 184.6, respectively). Vaginal tissue TFV concentrations were significantly positively correlated with vaginal epithelial thickness, whereas vaginal tissue TFV-DP concentrations were positively correlated with density of vaginal CD4^+^ and CD8^+^ immune cells.

**Conclusions:**

The state of the cervicovaginal mucosa has a significant impact on local and systemic PK of a topically applied microbicide.

## Abstract 2

Supplemental Digital Content is Available in the Text.

## INTRODUCTION

Microbicides are topical or systemic products that men and women can use to prevent human immunodeficiency virus type 1 (HIV-1) infection.^1^ Lead microbicide products use antiretroviral (ARV) compounds to prevent HIV-1 acquisition.^2^ In regions of the world where HIV-1 incidence is highest, women are disproportionately affected.^3^ Social, behavioral, and economic inequalities contribute to the increased vulnerability of women to HIV-1.^3^ However, biological cofactors including exposure to exogenous hormones (reviewed in Ref. 4) or fluctuations in endogenous hormones^5–9^ may be implicated in HIV-1 acquisition, transmission, and disease progression.

There are less data characterizing the interaction between endogenous hormones and systemic and local pharmacokinetics (PK) of microbicides. Much of the in vivo data on the interaction between ARVs and systemic exogenous hormones come from contraceptive and ARV drug–drug interaction studies (reviewed in Ref. 10). The effect of serum progesterone (P4) and serum estradiol (E2) on tissue PK of ARVs was modeled in primary cell lines.^11^ Others describe differences in PK and mucosal drug transporters in lower genital tract explants from premenopausal (PRE) versus postmenopausal (POST) women^12,13^ and in endometrial biopsies during the follicular (FOL) versus the luteal (LUT) phases of the menstrual cycle.^14^ In monkeys, there is a difference in the mucosal susceptibility to simian/human immunodeficiency virus infection between the FOL and the LUT phases of the menstrual cycle.^15,16^

Serum E2 and P4 concentrations are significantly different among PRE versus POST women^17^ and offer an opportunity to study the potential effects of endogenous hormone concentrations on ARV PK after in vivo use. We previously showed that the lower genital tract of PRE and POST women differ significantly, particularly in factors implicated in early mucosal HIV-1 acquisition, such as vaginal immune cell density and histology.^18^ These cofactors are also believed to be important in local ARV PK. We are not aware of studies that investigate local and systemic PK of topical microbicides in healthy PRE versus POST women and after POST women use in vivo topical E2 therapy.

The primary objective of this study was to describe and compare the systemic and local PK of tenofovir (TFV) in healthy PRE and POST women and POST women after topical E2 treatment, after using TFV 1% vaginal gel. We also correlate local PK parameters with mucosal end points obtained from these 2 cohorts. Our hypothesis was that these cohorts would have significantly different systemic hormonal profiles and local mucosal environments, and that this would result in differences in TFV PK end points.

## METHODS

### Clinical Study

The CONRAD A12-124 protocol was approved by the Eastern Virginia Medical School Institutional Review Board (13-02-FB-0017) and registered with ClinicalTrials.gov (#NCT01810315). All study volunteers provided written informed consent before any study procedures. We enrolled healthy, HIV-1 negative, non-pregnant PRE (n = 20) and POST (n = 20) women, who reported no use of exogenous hormones, had no evidence of reproductive tract infections such as bacterial vaginosis (BV) (Nugent score 7–10) or yeast vaginitis or sexually transmitted infections, and did not use tobacco or vaginal products (eg, douches, nonoxynol-9 spermicides). PRE participants reported regular menstrual cycles. Before product use, normal ovulatory status in PRE women was confirmed by a LUT phase P4 level of 3 ng/mL or higher. POST women had ceased menstruation for 12 months or longer, had no contraindications to vaginal E2 therapy, and a serum follicle stimulating hormone level of 20 mIU/mL or higher.

### Study Design

The study visits are outlined in Table 1. We sampled PRE women between menstrual cycle days 5–10 for FOL phase samples (visit 4, V4) and menstrual cycle days 20–25 for LUT phase samples (visit 5, V5). Genital sampling visits for POST women (visit 3, V3 and visit 6, V6) were separated by 26–40 days to allow for genital tract biopsy sites to heal. We asked participants to refrain from vaginal intercourse 48 hours before each genital sampling procedure and for 5 days after cervicovaginal (CV) biopsies. We were unable to confirm the absence of vaginal prostate-specific antigen with point-of-care testing for the TFV gel during visits because TFV gel may interact with the assay.^19^ Laboratory staff evaluating end points viewed only the participant number and did not have access to participant age, cohort, visit type, or other identifying information. This article reports on data obtained from visits where TFV 1% gel was used (as above and bolded in Table 1). We previously reported on data obtained in this population from visits in the absence of TFV 1% vaginal gel [PRE visits 2 (V2) and V3, POST V2 and V5].^18^

**TABLE 1.**
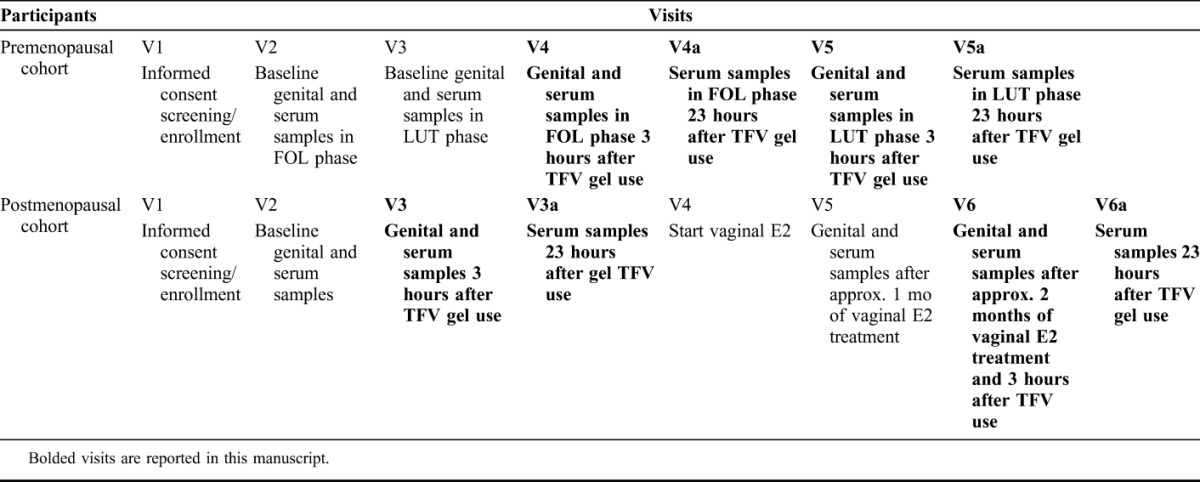
Table of Visits for the CONRAD A12-124 Study

### TFV Vaginal Gel Treatment

Participants inserted 1 applicator of TFV 1% gel (4 mL, 4.4 g) vaginally and repeated the dose 2 hours later to approximate the pericoital regimen used in phase 3 studies.^20,21^ They returned to the clinic 3 hours (±1 hour) after the second dose for CV biopsies and blood collection and then 23 hours (±1 hour) after the second dose for blood collection (Table 1). PRE women underwent 2 TFV 1% gel treatments in the FOL (V4) and LUT phases (V5). POST women underwent 2 TFV 1% gel treatments, at baseline (V3) and after using vaginal E2 therapy for approximately 2 months (V6).

### Estradiol Vaginal Cream Treatment

POST women used vaginal E2 (0.01%, 100 μg/g) cream (Estrace; Allergan, Rockaway, NJ) in the approved dosing regimen for the treatment of vulvovaginal atrophy. Specifically, they inserted 2 g of E2 cream every night for 14 days and then 1 g of E2 cream every other night for the duration of treatment. Genital sampling occurred approximately 1 month after commencing treatment (V5) and 2 months after commencing treatment in conjunction with TFV 1% gel use (V6).

At each visit, blood was drawn for plasma P4 and E2 levels and plasma TFV concentrations. At 3 hours after dosing visits, a CV aspirate for TFV concentration and 1 vaginal biopsy was obtained for the assessment of tissue concentrations of TFV, TFV-monophosphate (TFV-MP), TFV-diphosphate (TFV-DP), and competing nucleotides [deoxyadenosine triphosphate (dATP) and deoxycytidine triphosphate (dCTP)]. A second vaginal biopsy was obtained at these visits for the assessment of vaginal immune cells and characterization of other vaginal proteins, including drug transporters.

### Assessment of Plasma and Tissue PK Parameters and Endogenous Nucleotides

Vaginal tissue biopsies were collected in sterile cryovials and immediately flash frozen in liquid nitrogen. Whole blood was collected in a 3-mL K-EDTA Vacutainers and centrifuged at 4°C for separating plasma. Tissues, CV aspirate, and plasma were all stored at −80°C and then shipped as 1 batch under frozen conditions to the University of North Carolina Center for AIDS Research, Clinical Pharmacology and Analytical Chemistry Core for analysis.

All analytes were quantified by liquid chromatography–and tandem mass spectrometry (LC-MS/MS) assays, with a precision and accuracy of 15%. TFV was extracted from plasma samples with the isotopically labeled internal standard (IS) ^13^C_5_-TFV and quantified with a dynamic range of 0.25–250 ng/mL, as previously described.^22^ CV aspirates were diluted with 0.9% sodium chloride, and 50 μL of diluted sample was mixed with 150 mL of methanol containing ^13^C_5_-TFV IS. After vortex and centrifugation, the extract was evaporated to dryness, then reconstituted with 1 mM ammonium phosphate (pH 7.4), and TFV was analyzed under the same LC-MS/MS conditions as the plasma analysis with a dynamic range of 2–5000 ng/mL.

Tissue biopsies were homogenized in 70:30 acetonitrile:1 mM ammonium phosphate (pH 7.4) then extracted for TFV, TFV-DP, dATP, dCTP, and TFV-MP with the following isotopically labeled IS: ^13^C_5_-TFV; ^13^C_5_-TFV-DP; ^13^C_10_,^15^N_5_-dATP; ^13^C_9_,^15^N_3_-dCTP; and ^13^C_5_-adenosine 5′-diphosphate, respectively. After vortex and centrifugation, extracts were evaporated to dryness then reconstituted with 1 mM ammonium phosphate (pH 7.4). TFV, TFV-DP, dATP, and dCTP were analyzed, as previously described.^23^ TFV-MP was analyzed using anion exchange chromatography on a Thermo BioBasic AX (50 × 2.1 mm, 5 μm particle size) analytical column and detected under positive ion electrospray conditions on an AB Sciex API-5000 triple quadrupole mass spectrometer. The dynamic range was 6–6000 ng/mL homogenate for TFV; 0.3–300 ng/mL homogenate for TFV-DP; and 0.1–100 ng/mL homogenate for dATP, dCTP, and TFV-MP.

### Analysis of Immune Cells and Drug Transporters in the Vagina

For immunohistochemistry analyses, each vaginal biopsy was placed in 10% neutral buffered formalin for 24–48 hours and processed as per our existing protocol.^24^ Staining for drug transporter proteins was performed using specific monoclonal antibodies against MDR1, clone JSB‐1 (catalog #MAB4120; Millipore, Billerica, MA), MRP2 clone M2III‐5 (catalog #MC267; Kamiya, Seattle, WA), MRP4 clone 1B2 (catalog #H00010257‐M03; Novus Biologicals, Littleton, CO), and OAT1 (catalog #AB118346; Abcam, Cambridge, MA). The relative expression of transporters was scored manually based on their distribution and intensity of staining in the vaginal mucosa under the microscope (Nikon E-800).^25,26^ The scoring ranged from 0 to 3 based on the intensity of staining from none (0), low (1), medium (2), and intense (3). The scoring was performed by a single histopathologist (NC), with confirmation of counts in a subset of tissues by a histopathology assistant. The integrated optical density of E-cadherin and tissue density and phenotype of vaginal immune cells were characterized as previously described.^18^

Statistical analyses were performed with SAS version 9.3 (Cary, NC). Descriptive statistics included mean, median, and SD for continuous variables and frequencies and percentages for categorical variables. We compared continuous end points from PRE and POST cohorts using an independent samples *t* test for normally distributed data or Wilcoxon–Mann–Whitney test for nonnormally distributed data. Normality of data sets was tested using the PROC univariate command in SAS and then the normal quantile plot distribution and the Shapiro–Wilk test statistic were examined. For categorical variables from independent samples, we used χ^2^ statistic or Fisher exact tests as indicated by the expected cell size. Paired comparisons using a paired *t* test or Wilcoxon signed-rank sum test were performed on difference variables [changes in PRE samples obtained in the FOL (V4) versus LUT phases (V5) and differences in POST samples obtained before (V3) and after vaginal E2 therapy (V6)]. Because the PK, vaginal pH, vaginal Nugent score, and immune cell data sets exhibited a log-normal distribution, log 10 transformation was applied to avoid violating the normality assumption. For data requiring log transformation, trends or significant findings were tested with both parametric tests for log transformed, normally distributed data and nonparametric methods for non-normally distributed raw data. Data sets of participant age, serum E2, serum P4, vaginal epithelial thickness, and number of vaginal cell layers were normally distributed. For the correlations between serum E2 and P4 and PK parameters, a Pearson correlation coefficient was calculated for normally distributed data and a Spearman correlation coefficient was calculated for non-normally distributed data. We performed multiple linear regression analyses for normally distributed data and significant variables found with simple correlation, using a backward elimination method. Statistical significance was determined at the level of alpha = 0.05.

## RESULTS

### Study Population

We screened and enrolled 26 PRE and 22 POST women for the study. Among the women enrolled, 20 PRE and 17 POST women attended all visits and represented the analysis population, as previously described (Supplemental Digital Content Figure 1, http://links.lww.com/QAI/B123).^18^ Supplemental Digital Content Table 1 (http://links.lww.com/QAI/B123) demonstrates expected differences between the PRE and POST cohorts at screening, as previously published.^18^

### Differences in Serum E2 and P4 Among PRE Versus POST Participants

For PRE participants, FOL phase sampling after TFV gel (V4) use occurred on menstrual cycle day 7.9 (±2.0 days), whereas LUT phase sampling after TFV gel (V5) use took place on menstrual cycle day 21.9 (±1.6 days). Among the PRE cohort, serum E2 concentrations in the LUT phase (V5) (95.0 ± 52.2 pg/mL) were lower than those in the FOL phase (V4) (128.9 ± 119.6 pg/mL), but not significantly different (*P* = 0.18). Serum P4 concentrations in the LUT phase (V5) (6.2 ± 4.4 ng/mL) were significantly higher than those in the FOL phase (V4) (0.6 ± 0.6 ng/mL) (*P* < 0.01). Two PRE participants both of whom had serum P4 concentrations obtained on menstrual cycle day 23 at V5 had serum P4 levels consistent with anovulation (0.336 and 0.341 ng/mL). The results were calculated with and without the inclusion of these 2 participants and did not change the overall conclusions. The results reported below include these 2 participants because there is a possibility that the single serum P4 determination missed the LUT P4 peak, given that both participants had ovulatory serum P4 levels during screening (V2).

Among the POST cohort, concentrations of serum E2 at V5 and V6, after vaginal E2 treatment for 1 versus 2 months, respectively, (28.7 ± 8.8 and 34.3 ± 11.7 pg/mL, respectively) did not change significantly from baseline before treatment (V3) (28.9 ± 11.9 pg/mL) (*P* value = 0.27). Similarly, serum P4 concentrations at V5 and V6 after vaginal E2 treatment (0.2 ± 0.1 and 0.2 ± 0.1 ng/mL, respectively) did not change significantly from baseline (V3) (0.2 ± 0.1 ng/mL) (*P* value = 0.93). As expected, at all sampling points, serum E2 and P4 in PRE women were significantly higher than that in POST women (all *P* values < 0.01).

### Comparison of Paired PK End points Obtained From PRE Women in the FOL (V4) Versus the LUT (V5) Phases

Consistent with our published data,^9,18^ among the PRE cohort, we found no significant differences in paired comparisons of any PK end point between the FOL (V4) and LUT phases (V5) of the menstrual cycle (all *P* values > 0.10, Supplemental Digital Content Figures 2a–2d, http://links.lww.com/QAI/B123). Therefore, when comparing end points among independent PRE and POST cohorts, we pooled the paired FOL phase (n = 20) and LUT phase TFV gel treatment concentrations (n = 20) from PRE participants (V4 + V5). Mean TFV concentration in plasma at approximately 23 hours after the second dose of TFV [visits 4a (V4a) and 5a (V5a)] in both the FOL and LUT phases was <0.3 ng/mL (data not shown). In PRE women, there was a significant positive correlation (Spearman correlation coefficient 0.51, *P* value < 0.001) between tissue TFV and TFV-DP concentrations.

### Independent Samples Comparison of PK Endpoints Among POST Women at Baseline (V3), POST Women after Vaginal E2 Treatment (V6), and PRE Women in the FOL (V4) and LUT (V5) Phases

Table 2 demonstrates that local TFV concentrations were significantly lower in POST women at baseline (V3). Despite this, POST women efficiently converted TFV to TFV-DP, resulting in similar concentrations of the active metabolite, TFV-DP, in all cohorts and significantly higher molecular ratios of TFV-DP to TFV in POST women. Once POST women used topical E2 for approximately 2 months (V6), their PK parameters were similar to those measured in PRE women, except for significantly lower levels of TFV-MP (*P* value < 0.01, Table 2). The differences in vaginal tissue TFV concentrations remained significant between POST women at baseline and PRE women even after controlling for differences in vaginal pH and Nugent score between the cohorts (both *P* values < 0.01).

**TABLE 2.**
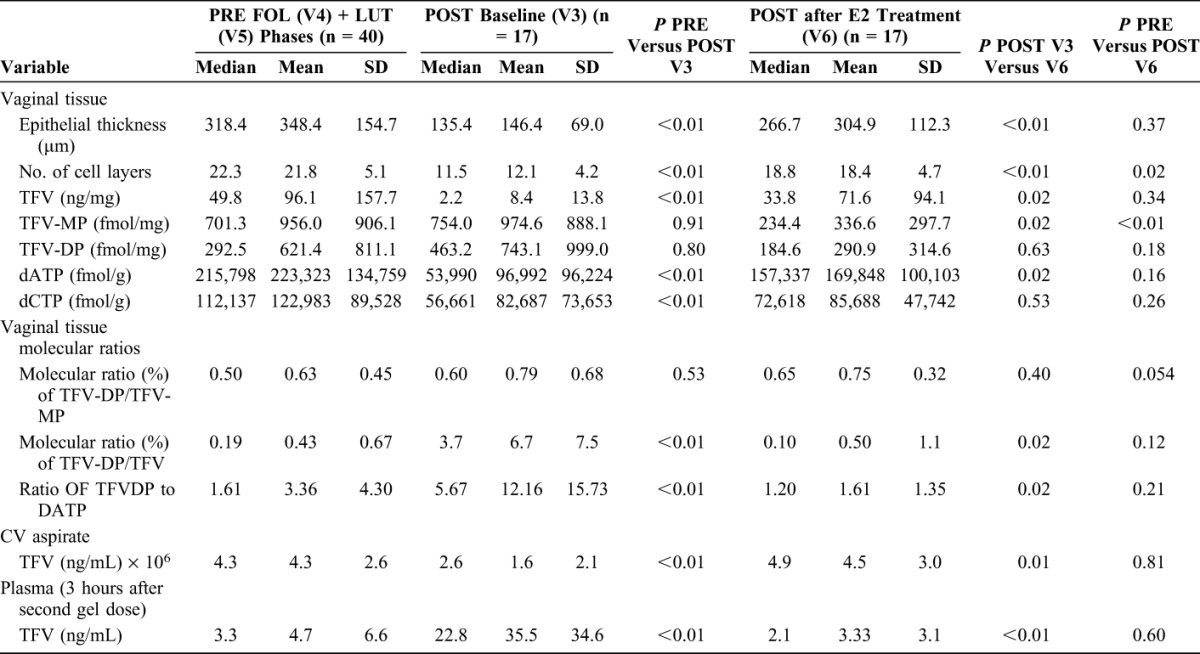
Differences in Vaginal Histology and TFV PK Endpoints Among PRE Women in the FOL (n = 20) and LUT (n = 20) Phase Combined Versus POST Women (n = 17) at Baseline Versus POST Women After Approximately 2 Months of Topical E2 Therapy

### Compairson of Paired PK Endpoints Among POST Women before (V3) and after Vaginal E2 Treatment (V6)

There were significant differences in most (10 of 12) of the local and systemic PK end points in paired comparisons of samples obtained from POST women before (V3) and after approximately 2 months of topical vaginal E2 treatment (V6) (Table 2 and Supplemental Digital Content Figs 2a–2d, http://links.lww.com/QAI/B123). Mean plasma TFV concentrations in POST women were low (<2 ng/mL) 23 hours after the second dose of TFV, both before [visit 3a (V3a)] and after topical E2 therapy [visit 6a (V6a)] and were not statistically different (multiple comparison *P* value > 0.05, data not shown). There were no significant correlations between vaginal tissue TFV and TFV-DP concentrations among POST samples obtained at baseline (V3) or after vaginal E2 treatment (V6) or when both visits were combined (all correlation coefficients <0.47, all *P* values >0.05, data not shown).

### Concentrations of Vaginal Tissue Endogenous 2′deoxynucleotide Analogs dATP and dCTP in PRE Versus POST Women

Table 2 and Supplemental Digital Content Figures 3a and 3b (http://links.lww.com/QAI/B123) demonstrate that both mean dATP and dCTP concentrations are significantly lower among POST women before E2 therapy (V3) compared with PRE (V4 + V5) and POST women after vaginal E2 treatment (V6). POST women have significantly higher TFV-DP to dATP molecular ratios.

### Correlation of Tissue PK Concentrations With Vaginal Immune Cells, pH and Histology in all Participants at all Visits

**FIGURE 1.**
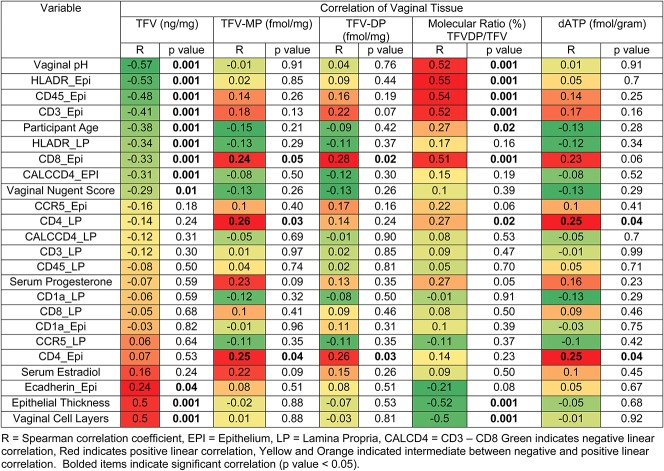
Heat map of simple linear correlations between vaginal tissue TFV and TFV-DP concentrations and the molecular ratio of TFV-DP to TFV with mucosal end points of all premenopausal and postmenopausal participants at all TFV gel use visits. EPI, epithelium; LP, lamina propria; R, Spearman correlation coefficient; CALCD4, CD3–CD8. Green indicates negative linear correlation, red indicates positive linear correlation, and yellow and orange indicate intermediates between negative and positive linear correlation. Bolded items indicate significant correlation (*P* value < 0.05).

In a previous publication,^18^ we reported differences in mucosal end points, including vaginal tissue immune cells, vaginal histologic parameters, and vaginal pH and Nugent score^27^ between the PRE and POST cohorts. To increase the range of endogenous hormone concentrations, samples from all TFV gel use visits [PRE FOL (V4) and LUT (V5), POST baseline (V3) and after E2 treatment (V6)] were pooled to determine whether significant linear correlations existed between local tissue concentrations of drug, serum hormone concentrations, and mucosal end points previously reported.^18^ Figure 1 shows that, in general, tissue TFV concentrations were significantly and positively correlated with epithelial histologic parameters including epithelial thickness and number of cell layers and the integrated optical density of E-cadherin. TFV concentrations in vaginal tissue significantly decrease with increasing density of vaginal immune cells and decreasing epithelial thickness (Fig. 1). Finally, vaginal environment end points such as vaginal pH and Nugent score were also significantly and negatively associated with tissue TFV concentrations, but had no effect on the active metabolite, TFV-DP. The molecular ratio of TFV-DP to TFV significantly increased with increases in vaginal pH. TFV-DP tissue concentrations were significantly correlated with vaginal epithelial immune cell density (in particular, CD4 and CD8 cells) (Fig. 1).

Multiple linear regression analyses were performed to predict vaginal tissue concentrations of TFV, TFV-MP, and TFV-DP and the molecular ratio of TFV-DP to TFV with variables that were significant in simple linear regression, adjusting for covariates. Vaginal tissue TFV concentrations 3 hours after topical application of TFV gel were significantly associated with vaginal epithelial thickness (R^2^ = 0.15, *P* value < 0.01); tissue TFV-MP concentrations were significantly associated with the density of CD4^+^ cells in the lamina propria (R^2^ = 0.26, *P* value < 0.01); and vaginal tissue TFV-DP concentrations were significantly associated with the density of CD8^+^ cells in the epithelium (R^2^ = 0.10, *P* = 0.01). Vaginal tissue dATP concentrations were associated with the density of CD4 cells in the lamina propria (R^2^ = 0.15, *P* = 0.049). Finally, vaginal pH and density of HLADR+ (activated) cells in the epithelium, together, were the parameters that best predicted the molecular ratio of TFV-DP to TFV in vaginal tissues (R^2^ = 0.48, *P* value < 0.01).

### Drug Transporters in Vaginal Tissue of PRE and POST Women

**FIGURE 2.**
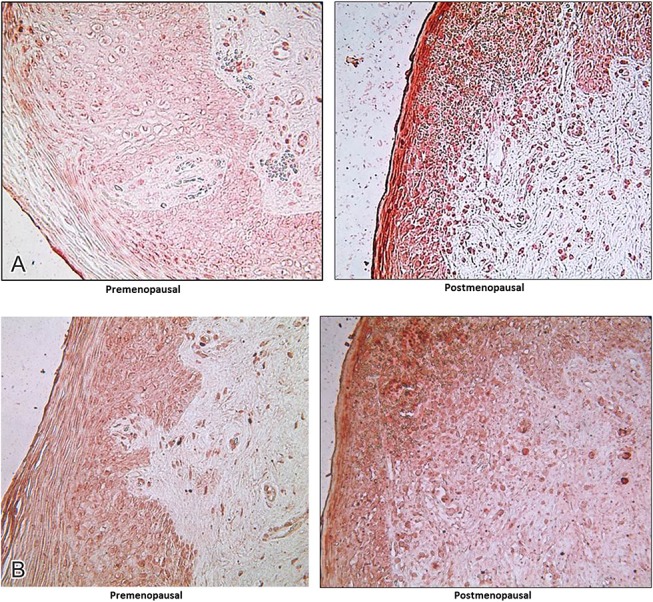
A, MRP-2 transporters in Ectocervix of PRE LUT phase versus POST at baseline. B, MDR-1 transporters in Ectocervix of PRE luteal versus POST at baseline.

OAT-1 was not present in the vaginal tissue epithelium or lamina propria of any participant evaluated. We also evaluated MRP2, MRP4, and MDR1 efflux transporters in both the vaginal epithelium and lamina propria. POST women have significantly higher density of drug transporters in the epithelium compared with PRE women. After E2 therapy, there was a significant decrease in the density of MDR1 in the vaginal lamina propria and MRP2 in both the vaginal epithelium and lamina propria in POST women. (Table 3, Figs. 2A, B).

**TABLE 3.**
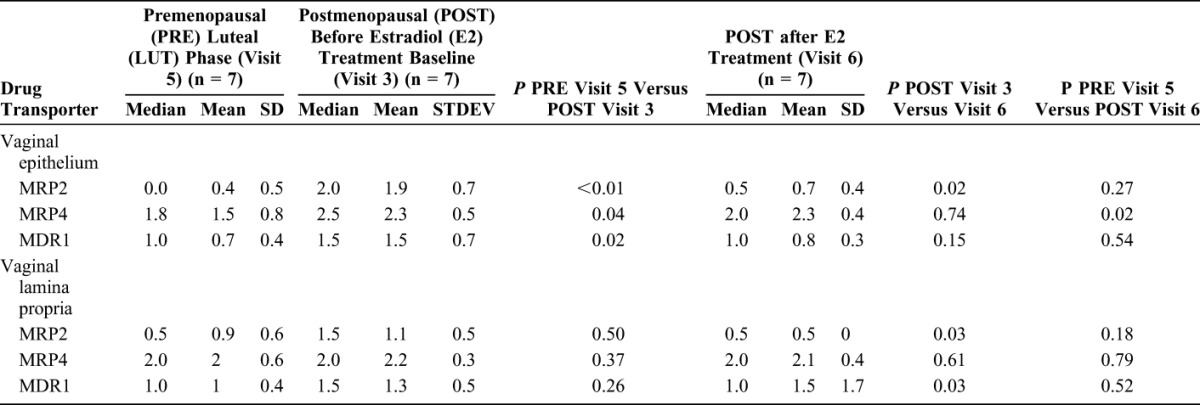
Differences in the Semi-Quantitative Density of Vaginal Tissue Drug Transporter Proteins Among a Subset of 7 Premenopausal and 7 Postmenopausal Participants

## DISCUSSIONS

We report significant differences in mucosal concentrations of TFV, TFV-MP, TFV-DP, endogenous nucleotides, and drug transporters after topical application of TFV 1% vaginal gel between PRE and POST women. POST women at baseline have lower TFV in CV tissue and CV aspirate but similar TFV-DP concentrations, leading to a higher tissue TFV-DP/TFV molecular ratio. Addition of E2 in POST women returned local and systemic concentrations of all end points, except TFV-MP, to levels similar to PRE women in the FOL and LUT phases, supporting that exogenous hormones also influence PK of topical TFV. Tissue concentrations of TFV-DP, the active intracellular metabolite, were similar in all groups at the time of sampling, reflecting differences in the efficiency of mucosal conversion among the reproductive states. Unlike PRE women, POST women had a nonlinear relationship between tissue TFV and TFV-DP concentrations. This demonstrates that POST women efficiently converted low concentrations of TFV into levels of TFV-DP that were similar to PRE women in the FOL and LUT phases. In accordance with in vitro models using CV explants,^28^ we found that endogenous nucleotides, dATP and dCTP, were lower in POST baseline tissues, leading to a higher TFV-DP/dATP ratio in POST women, which would endow TFV with higher activity. No significant differences were observed in FOL versus LUT samples in PRE women.

Plasma TFV concentrations in all participants, 3 hours after topical therapy, were 10–100 times lower than that seen after oral therapy.^29–31^ Although unlikely to be clinically significant, mean plasma concentrations in POST women at baseline were approximately 10 times higher than those after 3 hours of sampling. We attribute this to the significantly thinner vaginal epithelium found in the POST cohort before E2 treatment.^18^ In POST women, before E2 treatment, TFV had to diffuse through significantly fewer vaginal cell layers to enter the systemic compartment. Approximately 23 hours after topical treatment (“a” visits, Table 1), plasma TFV concentrations were similar between all groups and were 100 times lower than that seen after oral dosing.^29–31^ Local PK concentrations were not correlated with the systemic hormonal state (measured by serum E2 and P4, Fig. 1). By performing detailed mucosal and PK sampling in both PRE and POST women, cohorts that have different mucosal environments, we correlated local concentrations with several mucosal end points believed to be involved in TFV metabolism and mechanisms of early HIV-1 acquisition.^9,18^

We found no difference in any PK end points between paired samples obtained in the FOL versus the LUT phases among PRE women. In most cases, FOL and LUT sampling in PRE women occurred in the same menstrual cycle, separated by at least 10 days. We previously reported that CV biopsies are well healed in PRE women within 7–10 days.^32^ Our findings of no differences between systemic and local PK in the FOL and LUT phases support that this sampling frequency provided a sufficient washout period. Our study is the first study to compare local PK of a topically dosed ARV based on the menstrual cycle phase. Others found no differences in systemic or mucosal ARV concentrations among HIV-1–infected^33^ or HIV-1–uninfected^34^ women taking oral ARVs who were sampled throughout the menstrual cycle.

The concentration of TFV in tissue was positively correlated with the thickness and the integrity of the epithelium, which supports that the CV epithelium represents a reservoir for topically administered TFV.^11^ TFV tissue concentration was significantly negatively correlated with vaginal pH and Nugent score. This could be due to the recently reported effect of BV-associated microbiota and their ability to take up and degrade TFV in the CV fluid^35^ and is consistent with a recent study in PRE women who used either TFV 1% vaginal gel or TFV vaginal film daily for 6 days.^36^ Although clinical BV was an exclusionary criteria in this study, we found that microbiota changed in women during the study,^18^ and POST women are known to have more anaerobes in their vaginal microbiome.^37^ Topical administration of E2, which is known to stimulate both epithelial proliferation and has a beneficial effect on lactobacilli,^18,38^ resulted in increased concentrations of TFV at V6 in POST women, with levels similar to those of PRE women. Despite the effect of epithelial thickness, vaginal pH, and Nugent score on TFV tissue concentrations, all cohorts had similar concentrations of TFV-DP. Microflora-associated end points (vaginal pH and Nugent score) were not linearly correlated with concentrations of this important active metabolite. This is in contrast to the findings reported in the FAME 04 study^36^; however, this study did not use linear modeling for this correlation. Importantly, our participant cohort, products, and dosing regimen differed from the FAME 04 cohort and this likely contributes to the differences noted in the findings. We found that increases in vaginal pH were correlated with increases in the molecular ratio of TFV-DP to TFV; specifically, for every 0.52 increase in vaginal pH, the TFV-DP to TFV ratio increased by 1%. There is a keen interest in determining how vaginal pH and vaginal microbiota impact ARV PK,^35^ and these data provide insights into the interaction between the mucosa, endogenous hormonal state, exogenous hormone effects, and ARV PK.

Lower concentrations of TFV in tissues of POST women at baseline may also be due to an increase in efflux transporters such as MRP2 and MRP4 known to participate in TFV efflux from epithelial cells in the kidney.^39–42^ Drug transporter mRNA and protein expression has been described in explants from the uterus^43^ and the lower and upper female reproductive tract (FRT).^44^ Our in vivo data add to the existing explant and cell culture models which support that differences in endogenous hormones and use of exogenous hormones influence drug protein binding and drug transporter activity (reviewed in Ref. 45). The transporters MPR2, MRP4, and OAT1 are implicated in TFV disposition,^39–42^ and therefore, we characterized the protein expression of these transporters.^12,46^ Tenofovir disoproxil fumarate is a substrate for the MDR1 transporter.^47^ Tenofovir disoproxil fumarate is used both orally for HIV-1 prevention and is in the pipeline for topical prevention, and therefore, we also described this transporter in vaginal tissues. Consistent with other investigations of TFV-related drug transporters in the lower genital tract explants, we found that protein expression of the uptake transporter OAT1 in the vagina was low to absent.^12,48–50^ Our collaborators previously reported differences in vaginal, cervical, and colorectal drug transporters based on menopausal status in ex vivo tissue explants.^12^ Specifically, median mRNA expression of MDR1 in vaginal explants decreased by 17% in POST compared with PRE women and was negatively correlated with age.^12^ Using primary trophoblasts, others modeled the effects of E2 and P4, growth factors and cytokines on mRNA, and protein expression of MDR1, MDR3, BCRP, and MRP1 in vitro.^25^ In addition to our work, the only other in vivo characterization of the effect of endogenous hormones on drug transporter expression showed that MDR1 transporter mRNA expression in the endometrium varies significantly with the menstrual cycle in PRE women (n = 36).^14^

In spite of the differences in TFV levels in PRE and POST women, our data show similar concentrations of TFV-DP, the active metabolite which results from 2 intracellular phosphorylations of TFV.^1,51^ TFV-DP was positively associated with the epithelial density of immune cells, in particular CD4 and CD8 cells. We found that the conversion of TFV to TFV-DP is less efficient, with significantly lower molecular ratios of TFV-DP to TFV in an E2-replete environment of PRE women when compared with POST women at baseline. This is in contrast to our collaborator's cervical explant studies which showed significantly more efficient conversion of TFV to TFV-DP in explants from PRE women compared with POST women and significantly lower concentrations of TFV-DP in POST explants.^13^ Animal studies found mRNA levels of 5′ nucleotidases, the enzymes which phosphorylate nucleotide analogs such as TFV to TFV-DP, peak at estrus.^52^ In vitro studies show that these enzymes are present in FRT, with variable expression in the upper and lower FRT.^11^ However, mRNA expression not only varied with time of exogenous hormone exposure, but also with FRT cell type.^11^ This makes comparison of our in vivo findings to animal, in vitro cell, or explant tissue simulations difficult.

POST women at baseline had significantly lower levels of competing endogenous 2′deoxynucleotide analogs. Endogenous nucleotides have not previously been characterized in in vitro work examining the effect of hormones on ARV PK.^11^ Concentrations of these endogenous substrates proportional to TFV-DP or emtricitabine triphosphate affect the probability that HIV-1 reverse transcriptase will incorporate the drug molecule to inhibit viral replication.^51^ Higher molecular ratios of the active drug metabolites relative to endogenous nucleotides may be a marker of higher drug potency.^51^ Once POST women used TFV vaginal gel after 2 months of in vivo E2 therapy at V6, all of their local (except TFV-MP) and systemic PK end points were similar to the PRE cohort. As outlined above, using in vitro primary cell lines and explants, others found that in vitro E2 and P4 treatment of FRT cells altered enzyme expression and function, and hypothesized that in general, the efficiency of TFV to TFV-DP conversion would increase in the FRT with E2 exposure.^11^ Our colleagues also found that in explants, the percentage of TFV that was converted to TFV-DP was significantly higher in PRE explants compared with POST, treated in vitro with TFV-active pharmaceutical ingredients.^13^ On the contrary, we found that the molecular ratio of TFV-DP/TFV in vaginal tissue was highest in POST women at baseline in an E2-deficient state, and molecular ratios of TFV-DP to TFV were significantly reduced in E2-replete environments, after POST women received exogenous E2 and in PRE women. The increased TFV-DP/TFV ratio in POST women at baseline may be due to increased conversion of TFV by activated (HLA-DR+) immune cells together with accelerated efflux of residual TFV because of increased transporter density.^18^

The strength of this study resides in the fact that we followed a well-screened and well-characterized group of healthy PRE and POST women and performed detailed mucosal PK and mechanistic evaluations. Of note, this is the first study, in which the effect of in vivo E2 topical therapy on topical ARV microbicide PK and end points correlated with changes in PK was evaluated. Although we were able to retain women over a multi-month course of microbicide use and genital sampling, we acknowledge that the variability in the PK end points is high and this relatively small exploratory study could be affected by a type II error, particularly in terms of the TFV-DP concentrations. Although TFV 1% vaginal gel failed to demonstrate efficacy in 1 of 2 Phase 3 prevention trials,^21^ TFV remains an important component of the topical prevention pipeline. The wealth of PK modeling for HIV-1 prevention efficacy for TFV 1% vaginal gel in non–human primate models^53,54^ and efficacy benchmarks for both HIV-1^55,56^ and HSV-2^57^ in the CAPRISA cohort make it an important product to study and examine mucosal differences in PRE and POST women.

We obtained genital tract samples 3 hours after the second dose of TFV vaginal gel to replicate an episodic regimen used in previous phase III trials of TFV vaginal gel.^20,21^ It would be ideal to obtain multiple tissue biopsies at many time points after topical vaginal application of ARVs, but this entails multiple invasive procedures. The PRE and POST cohorts could also be subdivided into multiple sampling times, but this would reduce the subgroup size. Sampling in the FOL and LUT phases of the menstrual cycle among PRE women was based on the menstrual cycle day. It would have been more accurate to base these samplings on urinary lutenizing hormone surges to better pinpoint ovulation, however, this was not feasible in this study. Despite the requirement that all PRE participants demonstrate a LUT phase serum P4 of 3 ng/mL or higher before enrollment, we had 2 PRE participants with single serum P4 concentrations consistent with anovulation during the study.

In conclusion, the endogenous hormonal milieu and local mucosal environments of PRE and POST women differ substantially, and these data support that changes in the CV environment affect the local and systemic PK of a topically applied microbicide. Based on several mucosal environmental factors, POST women are at enhanced risk of acquiring HIV-1.^18,58–60^ This appears to be compounded by low levels of TFV in CV fluids and tissues after topical application of TFV 1% gel. However, higher efficiency in TFV conversion leading to high levels of TFV-DP, an active metabolite, in conjunction with reduced levels of competing endogenous nucleotides, provides the basis for an effective intervention based on topical delivery of TFV. It is unclear whether this advantage would also be seen after oral administration of TFV, such as in the case of oral emtricitabine/TFV disoproxil fumarate, but it is clear that mucosal environmental factors affect both susceptibility to HIV-1 and efficacy of prevention strategies such as those based on ARVs.

## Supplementary Material

SUPPLEMENTARY MATERIAL
