# Supplementary material for: Differences in Local and Systemic TFV PK Among Premenopausal Versus Postmenopausal Women Exposed to TFV 1% Vaginal Gel
Source: J Acquir Immune Defic Syndr. 2018 Feb 7;78(1):82–92. doi: 10.1097/QAI.0000000000001648 (PMC5902131; doi:10.1097/QAI.0000000000001648)
Supplement: SUPPLEMENTARY MATERIAL [file qai-78-82-s001.pdf]

Supplemental Figure 1: Premenopausal and Postmenopausal Populations

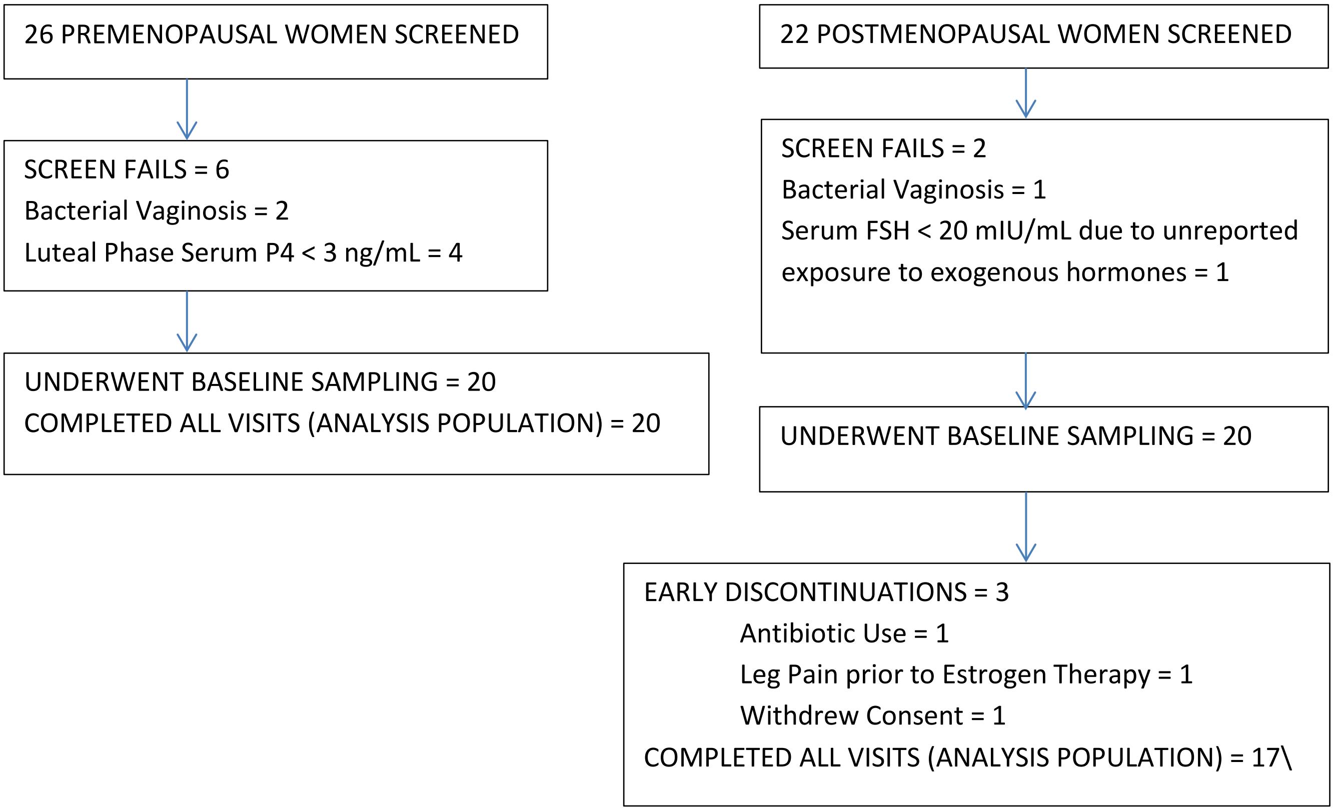

Supplemental Table 1: Analysis Populations

Variable	Premenopausal (PRE) Cohort (n = 20)			Postmenopausal (POST) Cohort (n = 17)			p value
	N	Mean	STD	N	Mean	STD	
Age (Years)	20	35.2	6	17	60.6	9.3	< 0.01
Years of Education	20	14	1.5	17	13.8	2.2	0.89
GRAVIDITY	20	3.1	1.6	17	3.2	3.2	0.40
PARITY	20	2.5	1.4	17	2.4	1.9	0.70
Body Mass Index (kg/m2)	20	31.9	9.8	17	29.3	6.2	0.53
Systolic Blood Pressure (mm Hg)	20	115.3	18	17	129.8	20.1	0.02
Diastolic Blood Pressure (mm Hg)	20	71.9	9.7	17	76.4	13.4	0.09
Vaginal pH at Screening (Visit 1)	20	4.3	0.3	17	5.3	0.7	< 0.01
Vaginal Nugent Score at Screening (Visit 1)	20	1.3	1.4	17	4.1	1.9	< 0.01
Race	N	% of Group		N	% of Group		p value
Non-Hispanic	17	85		17	100		0.15
Hispanic	3	15		0	0		
Ethnicity	N	% of Group		N	% of Group		p value
American Indian or Alaskan Native	1	5		0	0		0.11
White	13	65		14	82		
Black or African American	6	30		3	18		
Partner Information	N	% of Group		N	% of Group		p value
Living with Sexual Partner	12	60		6	35		0.02
Not Living with Sexual Partner	5	25		1	6		
No Sexual Partner	3	15		10	59		

Supplemental Figure 2: Vaginal Tissue Concentrations of TFV, TFV-MP and TFV-DP obtained from Premenopausal (PRE) women in the Follicular (FOL) and Luteal (LUT) phases (Blue) and from Postmenopausal (POST) women at baseline (BASE) and after approximately 2 months of vaginal estradiol (E2) treatment (Red). All vaginal tissues obtained 3 hours post TFV gel dosing.

Fig 2a: Tissue TFV (ng/mg) in PRE and POST Cohorts

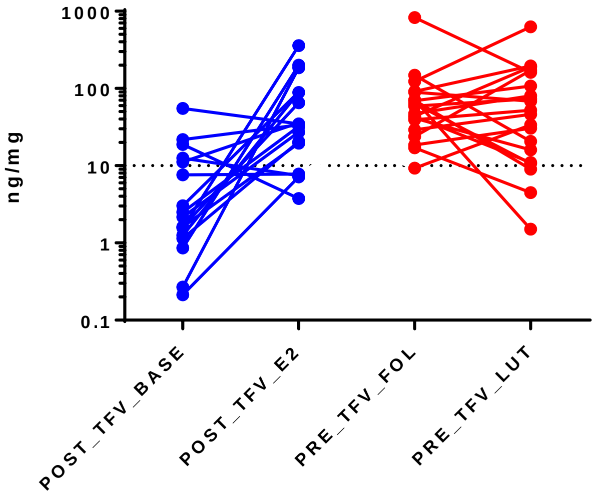

POST p value = 0.02
PRE p value = 0.82

Fig 2b: Tissue TFV-MP (fmol/gram) in PRE and POST Cohorts

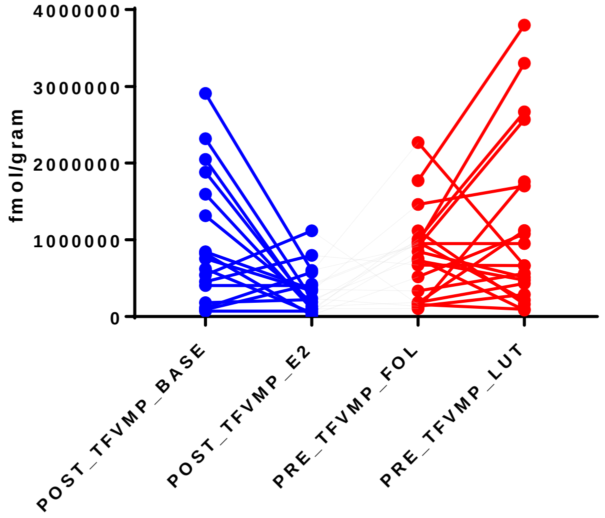

POST p value = 0.01
PRE p value = 0.15

Fig 2c: Tissue TFV-DP (fmol/mg) in PRE and POST Cohorts

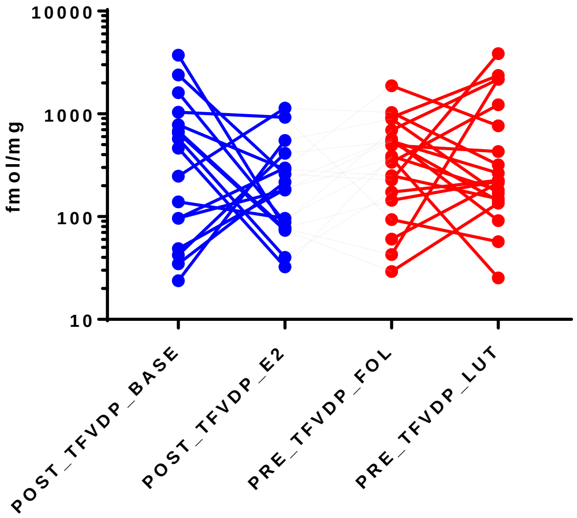

POST p value = 0.63
PRE p value = 0.50

Fig 2d: Tissue Molecular Ratio TFV-DP/TFV in PRE and POST Cohorts

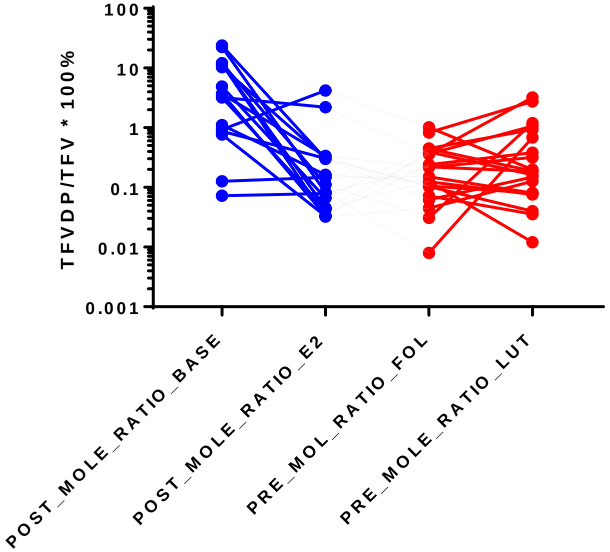

POST p value = 0.02
PRE p value = 0.10

Supplemental Figure 3: Endogenous nucleotide concentrations (fmol/gram) among Premenopausal (PRE) women (in Follicular (FOL) (Visit 4) and Luteal (LUT) (Visit 5) phases combined) (blue) versus Postmenopausal (POST) women at baseline (BASE) before (Visit 3) (red) versus after treatment with 2 months of topical estradiol (E2) cream (Visit 6) (green).

Figure 3a dATP in PRE vs. POST before and after E2 Tx

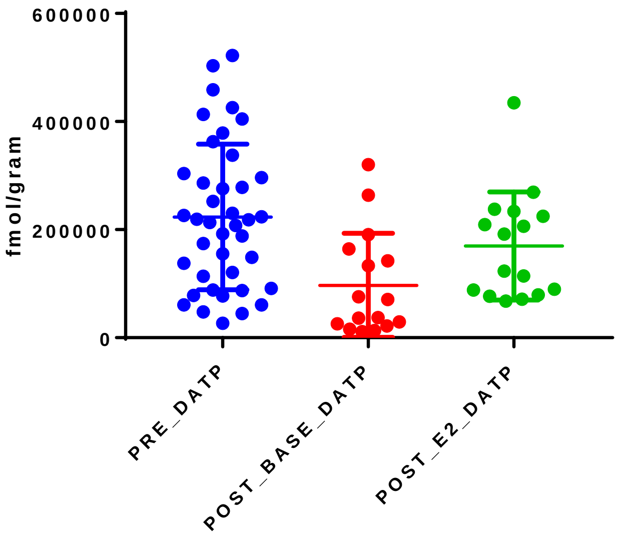

POST Baseline p value < 0.05

Figure 3b: DCTP in PRE vs. POST before vs after E2 Tx

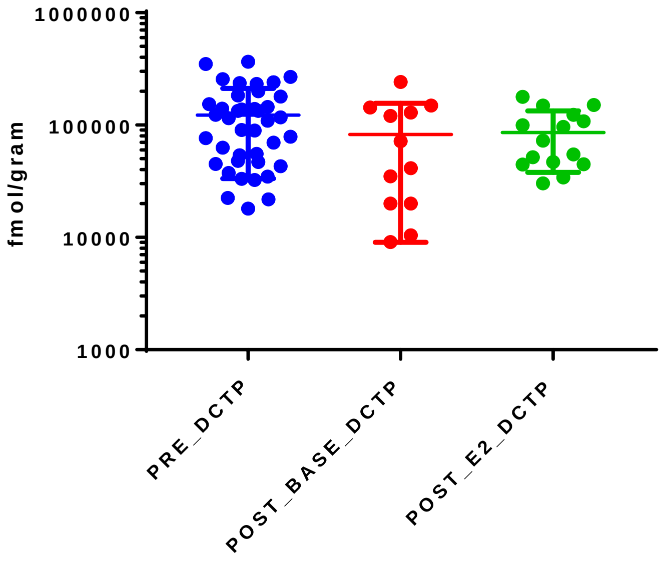

POST Base significantly lower than PRE p value = 0.02
